# Building adaptive self-healing systems within a resource contested environment

**DOI:** 10.1016/j.heliyon.2016.e00100

**Published:** 2016-04-29

**Authors:** Brian Phillips, Mark Blackburn

**Affiliations:** Department of Systems & Enterprises, Stevens Institute of Technology, United States

**Keywords:** Engineering, Computer science

## Abstract

Critical Software systems must recover when they experience degradation, either through external actors or internal system failures. There is currently no accepted generic methodology used by the software engineering community to design self-healing systems. Such systems identify when they require healing resources, and then change their own behavior to acquire and utilize these same resources. This study investigates using a design pattern to build such a system. It uses simulated robot tank combat to represent a challenge faced by an adaptive self-healing system. It also investigates how an adaptive system chooses different behaviors balancing its actions between healing activities, movement activities, and combat activities.

The results of this study demonstrate how an adaptive self-healing system utilizes behavior selection within a contested environment where other external actors attempt to deny resources to it. It demonstrates how a multi-system architecture inspired by cognitive science its behavior to maximize its ability to both win matches, and survive. This study investigates system characteristics such as how behaviors are organized and how computer memory is utilized. The performance of the adaptive system is compared with the performance of 840 non-adapting systems that compete within this same environment.

## 1 Introduction

This paper investigates building a self-healing adaptive system that uses a design pattern inspired by the neocortex of the mammalian brain. This pattern uses two separate behavior selection systems to choose behaviors based on the availability of data and the needed speed of the behavior change. This study compares two configurations for a hierarchy of behaviors by examining performance differences, and differences between the memory usages of each configuration.

The research team utilizes an extended RRobots simulation to provide an experimentation environment [1]. This is used to investigate the efficacy of different adaptation strategies based on machine learning techniques and a two-system behavior selection process. The novel contribution of this research is a demonstration of how such a design pattern could be used to build self-adaptive self-healing systems within a resource or memory constrained, competitive environment.

This paper uses the definition of self-healing systems put forward by [2]. It considers a self-healing system to be a specialized form of self-adapting system that can observe challenges to its system goals. Self-healing systems act to recover from degradation using resources within their own system's control, or within the environment.

Building self-adaptive systems is currently a task performed by highly skilled technologists and scientists, custom crafting a unique solution for a unique problem. This is because the skills and knowledge are too specialized and rare in the engineering community. A literature search for self-adaptive self-healing systems yields a number of examples supporting these claims. There is currently no general-purpose methodology of creating self-healing system using less specialized engineers.

This research uses a design pattern to codify the initial design stages of such a system, making that practice reproducible across a wider span of the engineering community. This effort presents a study of using such a design pattern approach with the goal of growing this into a stable methodology used and accepted by a broader community of system builders. A general approach for building self-healing self-adaptive systems expands the field of self-adaptive systems because such approaches will be reused across multiple efforts and projects. Reuse allows patterns to be altered, changed, and grown according to the pragmatic needs of system builders.

Self-adaptive systems change their behavior according to emerging opportunities or stresses within their environment. Self-adaptive systems typically possess a control loop that allows it to collect information about its internal state and the state of the environment. Such systems contain logic to analyze this information and represent the system state as a set of features that are used to inform current and future decisions. These decisions may include a behavior change, plan generation, or creating hypotheses. The control loop allows the self-adaptive system to act on these decisions. Such systems typically collect information on how the decision was made, and how the results of that decision affected the goals of the system. The foundations of engineering such systems remain an active research area [3].

A self-healing system seeks to identify points in time where it would benefit from healing activities and then changes its behavior to achieve this healing. It must detect a stimulus event that triggers the adaption while operating. Healing activities may involve exploiting additional resources, gaining idle time, performing background maintenance, or alerting external actors to potential risks to the system. Autonomic computing [4] systems use self-healing adaptation to reduce the level of human supervision and maintenance for that system.

Domain specific research has explored building self-healing systems across a variety of areas including internet service discovery [5], protective relay functions [6] in power systems, and the design of operating systems [7].

Other researchers have investigated using formal models and rule sets to create self-healing systems. The authors in [8] have researched building systems that utilize reference models external to a system. These reference models would act as a guide used by the system to adapt and heal. This approach is similar to run time verification techniques [9]. Minsky provides a method for understanding whether self-healing will be effective as well [10]. This approach uses an external assessment of the self-adaptive system to inform or trigger healing actions.

Research efforts have investigated building biologically inspired self-healing systems based on cell models [11]. Artificial immune systems [12] act to detect system vulnerabilities and overcome those with state changes and new behaviors. This effort adopts a similar approach. It uses an abstract biological system as a basis for the design of an information system.

This research is different than those noted previously; this approach and experimental prototype is based on a model documented within cognitive science literature. It uses a simplified cognitive model based on observations of the mammalian neocortex as a generic pattern for building the system. A design pattern based on a cognitive model provides a large degree of adaptation. Many self-adaptive systems use past experience to learn new adaptation strategies based on experience. They also use previous experience, heuristics, and rule-based systems to plan for possibilities, and then quickly adapt when one or more trigger events occur.

This study posits that a system based on cognitive decision processing rather than cellular adaptation is suited for larger distributed applications. Such a system selects from a set of behaviors to accomplish one or more goals. This becomes increasingly important when a system has multiple goals, and some system goals are more critical than others. Immune system goals seek to protect a system at all times from a variety of threats. Self-healing systems trade between executing their system goals and realizing an enhanced level of system health.

Many such systems inhabit the global technical environment. Adaptive information technology (IT) systems require routine maintenance and upgrades [13]. The U.S. Air Force is actively pursuing using self-healing techniques to improve system resiliency and recoverability in a cyber-combative environment [14]. The Defense Advanced Research Program Agency has taken this technique into the cyber-physical realm by beginning research into minimally invasive neurological implants that assist human patients [15]. Three other examples are listed below:

1. Fault detection systems identify when a trigger event occurs and evaluates the effect of that trigger upon the system. The system changes its state or behavior to address this fault [16].
2. A system checks its internal state against a reference model. The system changes its configuration or settings when its state lies outside of the boundaries set in that model [17].
3. System architectures use techniques inspired by biological systems to achieve an artificial immune system response [18].

[19] presents a component level description of different self-healing methods. Their paper organizes the many elements of self-healing into an ontological structure. It does not indicate any sense of a “best-practice” within the software engineering field. Essentially, designing self-healing systems continues to be a unique practice based as much on the backgrounds of the system builders as it is on an approach that has been adopted by practitioners as the standard.

This research effort extends these ongoing efforts by investigating a self-healing system within an environment with limited healing opportunities, and where an opponent seeks to dominate or deny access to these same resources. IT systems must execute maintenance within an environment populated by multiple critical customer transactions and requests. Air Force computer systems must react to cyber attack in a way that denies asset vulnerabilities to attackers, and still allows the use of those assets. Systems implanted in a living body must execute their function without harming the surrounding host and potentially survive the immune system activities of that same host. This research seeks to contribute to such efforts by providing an example of how engineers design and build such a system.

## 2 Background

Systems engineers and software engineers often benefit from using established design patterns when creating new systems [20]. These patterns present a number of well-understood approaches to solving different types of design problems. The Neocortex Adaptive Systems Pattern (NASP) [21] provides a useful starting point for building adaptive systems. This design pattern codifies an architectural approach used to build a decision system that uses short term and longer-term behavior selection approaches. This paper uses that pattern to investigate how a system decides how and when to execute self-healing actions.

This research regards a design pattern as a formalization of a software engineering approach that already exists. Indeed, the authors of this paper have seen many examples of different hierarchical models for building adaptive systems. Such examples are readily found in the fields of Deep Learning [22]. This paper defines a generic architecture composed of the primary components found within sample hierarchical learning system. This pattern defines a system architecture that is informed by these deep learning models, and influenced by neural and cognitive science observations. The cognitive science aspect loosely models the organization of a two-system decision system documented by Kahneman [23] and a hierarchical behavioral selection architecture.

The NASP natively supports using multiple types of decision systems with multiple time scales within an adaptive system. Kahneman [23] discusses the architecture of such a system and its implications on human decision making. The decision process within NASP uses a tree structure of possible adaptation decisions, inspired by direct neurological observations [24]. A system built using the NASP design approach compares these decisions against each other, either globally or within a more limited context. Behavior selection processes use numeric techniques such as Particle Filtering or Bayesian Classification [25] to select a single behavioral choice from a pool of candidates.

The NASP model solves many different types of behavior selection problems. Engineers specify adaptation decisions in a NASP based system design process. They specify decision nodes within a tree hierarchy to process information received from sensors and external sources. Those decisions may be directly created by software engineers or artificially generated using larger scale computational techniques. The NASP architecture is modeled on a biological system that is sufficiently generic to make decisions in multiple problem domains within the neocortex. [26] demonstrated this by examining different parts of primate brains. The tree-based architecture is found throughout the brain across different brain areas and functions.

The component architecture of NASP is a hierarchy of decision nodes. Each node in the NASP hierarchy is either a decision node that selects from a pool of possible behaviors, or a single behavior. Decision nodes can possess many children who are either candidate behaviors, or another decision node that optionally contains additional children. Child nodes can also be formed by decision nodes proposing many kinds of solutions or behaviors to address incoming stimulus values.

Information enters the system in the form of a stimulus signal or trigger event. Processing begins at the root node and flows down each branch of the tree. The decision nodes pass information to child nodes for processing. Then the decision node selects a best candidate behavior from among its children.

The example representation shown in Clip 1 has a root node shown by a triangle. That root node has children that addresses two types of problems, named Classifier A shown by squares, and Classifier B shown by circles. Those are decision nodes. Each of those problem types can be solved using a different set of stimulus values. Some of the classifiers use a limited set of information to make decisions more quickly. Hollow shapes represent these.

Some of the decision nodes use more stimulus information to arrive at a solution with higher accuracy, shown by the solid black shapes. The root node uses a tree search technique to select the best of all behaviors based on the most current information values stored within the information bus. Each decision node can contain a set of decision filters as well.

When a decision node seeks to select a behavior or action, it uses these decision filters to remove a set of least-optimal candidates. Heuristic algorithms allow the decision node to rapidly eliminate many of the optional children from being candidates for selection. Decision nodes compare the advantage of choosing one child solution over another based on the current stimulus and data stored within the information bus. This allows the decision node to choose a single candidate action to respond to incoming stimulus information. This approach fulfills the role of the particle filter.

Some decision nodes will receive inputs and consider short term information. Other decision nodes can execute detailed planning and processing over a longer time period. This particle filtering approach provides a multi-system way of integrating learning, behavior selection, and decision-making. A key feature of a NASP is that the decision nodes are orchestrated by feedback loops within a single branch, or across all branches of a NASP-based system.

This capability is represented in the architecture as a generic information bus. The information bus can be created from a blackboard [27] data system or a relational database. The information bus is shown in 1 as a large rectangle beneath the tree. Many decision nodes interact with the information bus. This is shown by the dotted arrows. Decision nodes either write information into the bus, or read it.

Decision nodes can use many approaches to dynamically adapt. They can use fixed rules, Bayesian statistics, Artificial Neural Networks, Genetic Algorithms, or inputs from an external system. The adaptation process consists of receiving stimulus information from a parent node, processing the information, storing the information for later use, and searching for additional information from a general bus of knowledge. Different decision nodes can add information to the information bus and make it available to another decision node anywhere in the system. When a triggering event arrives into the system, the decision node uses that information to select a behavioral response based upon the content of the triggering stimulus. In the context of a NASP, a trigger event is an event that causes a change in system behavior beyond the content of a data set. A stimulus event is any event that arrives into the system either from the exterior environment or interior component regardless of whether a behavior change occurs. Each layer in the architecture receives stimulus information. It selects a best response from its children. In some cases, a triggering event causes a decision node to dynamically generate children. In other cases, those child responses are chosen from a static list.

Past research efforts have demonstrated how a system built based on the NASP design pattern successfully adapt within a competitive environment [1]. Past research featured an adaptive system choosing from a pool of different behaviors while battling another within a fixed and constrained simulated two-dimensional battlefield. This study expands that research by adding a healing resource into the simulated battle. The opposing systems within the simulation must balance their primary function of defeating their enemy system with acquiring healing resources to enhance their survivability.

## 3 Hypothesis

This study demonstrates a self-adaptive system that shares an environment with another system, where these two systems compete for shared resources while attempting to degrade or destroy each other. The adaptive system will exploit opportunities to perform self-healing when advantageous. It does this while winning a majority of matches against non-adaptive systems with similar capabilities. The hierarchical architecture of adaptation decisions may contain one set of possible behaviors, or it may contain more than one set, each competing with the other. This study extends prior research on self-adaptive competing system with focus on investigating strategies for self-healing.

The objective of this study is to illustrate that adaptive systems outperform non-adaptive systems, and show that a hierarchical architecture decreases the system requirements (in the form of memory usage) with negligible impact on the final system performance.

This objective leads to the following hypotheses:

H1 – The performance of the self-adaptive system will exceed the average performance of the non-adaptive system.
H2 – The hierarchical architecture of the adaptive decision tree (also referred to as a branch topology) will not affect the outcome of these matches.

## 4 Materials & methods

This experiment is conducted using two competing agents that oppose each other within a simulated battlefield. One of those competing agents is a self-adaptive system capable of choosing its own behaviors based on a set of incoming stimulus signals. The other agent is a non-adaptive agent that uses a set of fixed behaviors. Each of these agents is referred to as a robot because the RRobots code represents competing agents as a robot tank. The adaptive tank is a tank agent that possesses every behavior that the set of all non-adaptive tanks possesses.

Clip 2 represents how robot tanks use behaviors within the RRobots simulation. Each non-adaptive tank has a specific set of behaviors that it uses, shown by solid triangles. These behavior sets include a single behavior choice for each of the different categories of behaviors, as discussed in Table 1. Each behavior in this pool of behaviors exists within one of the non-adaptive tanks. The non-adaptive tank selects a single behavior and does not change for the entire match. The adaptive tank chooses a single behavior from this same pool of behaviors, but it has the potential to change its behavioral choice at a later time. Clip 2 indicates a single behavior selection by a solid black line, and potential behaviors that could become this choice later by a dashed line.

The extended RRobots capability and environment includes a new type of agent called the Helicopter. The Helicopter enters the battlefield at specific times. It chooses a random location in the battlefield and moves to that location. Once it arrives, it pauses and then deposits a new resource named Cargo into the battlefield. Once the Cargo is deposited, the helicopter travels to the edge of the battlefield and leaves the match until its next scheduled arrival.

Cargo resources have no behavior. They exist within the battlefield at a specific location. When a robot tank moves to its location, the robot receives a healing score, improving its ability to receive damage without being destroyed. The Cargo is then removed from the battlefield.

Robot tank agents observe the moment when a Helicopter enters or leaves the battlefield and the location of the Helicopter when it is in the battlefield. They perceive when and where a Cargo resource is deposited into the battlefield environment. Tank agents may ignore the Helicopter and its newly deposited Cargo. They may change their behavior to attack the Helicopter and chase it from the battlefield. A tank may also attack a Cargo resource to destroy it, and thus deny the opponent an opportunity to heal.

Cargo, helicopters, and tanks accumulate damage by being hit by a simulated weapon. Each robot tank has a gun that fires shots. Shots travel the battlefield in a straight line from the tank until they exit the battlefield boundaries, or they are collocated with another agent and hit the target. Tanks control the angle of a turret, which in turn controls the angle of each shot fired. Each tank begins a match with a health score of 100. The simulation applies damage to a tank by reducing its amount of energy by the shot damage until an energy score of zero is reached. A zero energy score results in that tank loosing the match.

Energy scores are also reduced when a tank fires its gun. Every time it shoots, it reduces its survival ability because it uses energy to fire that gun. Tanks use different strategies to balance their fire power, damage expectation, and aiming.

Tanks use sensors to detect their opponent. The sensor detects an opponent by first sending a sensor pulse in a straight line, originating from the source tank. A sensor pulse that intersects another robot will cause the environment to trigger an event indicating the distance to the target and whether this is a tank, cargo, or helicopter. The environment triggers that event into the sensing tanks. Tanks use different strategies to rotate their radar and send sensor pulses.

Tanks use their sensor events to aim their simulated guns. The tank aims its gun by rotating a turret. Tanks use a number of strategies to aim their gun and fire. Opponent tanks move as well. Opponent tanks use strategies to decrease the number of shots that hit them, or increase the number returning shots that hit.

This research effort uses a vocabulary to describe how robot battles are organized. It uses the terms match, group, and tournament to describe the tiered structure of the experiment. Tanks compete using a set of battles within the simulated battle environment. A single battle is a ‘match’. Executing multiple matches between each tank type generates the probability of one tank winning against another. A ‘group’ is a set of matches. For example, a group can be composed of 100 matches between tank 1 and tank 2. Every combination of the five behavior types was used to define a non-adaptive tank that participated in a 100 match battle with the adaptive tank. The term ‘tournament’ describes a set of multiple groups that have a single unique tank combatant in common across all groups, and thus all matches.

RRobots uses the Ruby software language to implement agents. A software algorithm combines different parameters to create a population of 840 different types of non-adapting tank agents. Non-adapting agents begin the match with a single behavior selected for each strategy, which remain fixed through the entire match. Each non-adapting tank possesses a single value for each of the parameters. These parameters are shown in Table 1.

For example, a non-adapting robot could have a firing strategy of 1 where it expends less energy to fire at targets farther away, an aiming strategy of 0 where the tank uses dead reckoning to estimate where the target will be, a healing strategy of 0 where it ignores supplies, a movement strategy of 0 where it executes continuous circular motion, and a fire power level of 1.6 energy per shot.

These tanks are implemented using an object-oriented approach. Each non-adaptive tank class descends from a basis class that implements each of the strategies found in Table 1. A software application combines these parameter values and creates new classes that descend from the basis class.

The RRobots simulation executes 100 matches between each of these tanks, resulting in a total of 70,560,000 matches. The study uses the results of those matches to determine the probability of one non-adapting tank defeating another non-adapting tank based on their strategy/parameter values. The adaptive-tank uses this probability information as a basis for selecting behaviors once the identity of its opponent is known.

The adaptive tank is a tank agent that possesses every behavior that the set of all non-adaptive tanks possesses. It enters the match with a randomly selected behavior consisting of a firing strategy, damage level, movement strategy, aiming strategy, and healing strategy. The adaptive tank seeks to change its behavior with the goal of optimizing its win probability. An optimal behavior selection depends on type of opponent faced. The adaptive tank does not know the identity of its opponent at the start of the battle. As the battle progresses, the adaptive tank gathers evidence makes an assumption about the identity of its opponent, and chooses a behavior strategy based upon that assumption.

The adaptive tank periodically chooses a new behavior set based on the current assumption of opponent identity. Each time step allows an adaptive tank to observe the actions of its opponent and compute feature values based upon those actions. A set of tank battles that compete every non-adaptive tank type against the other 100 times computes a win probability table. This table shows the probability of a behavior for one tank type defeating the behavior of another tank type. The adaptive tank uses this table to select a best behavior.

This simulation approach is similar to that found in [1]. Tanks move within a two-dimensional battlefield. The match ends after a specified number of time steps resulting in a win if one tank is destroyed, or a draw if neither tank is destroyed. Each tank uses its sensors to detect an opponent. The tank aims its gun and fires shots using an energy level to inflict damage.

The simulation now contains a new aspect, self-healing. It also contains a set of self-healing behaviors. The self-healing aspect requires that the tank agent form an assumption about the healing strategies of its opponent. This assumption is based on these features:

1. Number of times an opponent shoots at and hits a Helicopter agent
2. Number of times an opponent shoots at and hits a Cargo resource
3. Number of times an opponent picks up a Cargo resource

This study also compares the performance of an adaptive tank with a one-branch behavior tree, to the performance of a system with a two-branch behavior tree as shown in Clip 2. The two-branch system uses two different methods to select a preferred behavior, and then merges those selections into a single behavior selection. This two-branch topology features one branch that chooses a behavior based on the strategies that represent non-healing actions such as movement, firing, aiming, and damage level. The other branch contains behaviors based on self-healing strategies.

Clip 3 shows a high-level object relationship diagram comparing these two topologies. The one-branch method requires 840 objects in memory to execute its function. The two-branch method requires 214 objects in memory composed of 210 combat behavior objects and 4 self-healing behavior objects. The two-branch method uses less computer memory.

### 4.1 Experiments

The study demonstrates H1 by executing 100 matches between the adaptive tank and every non-adaptive tank. The research team calculates a probability of the adaptive tank winning a match, and compares it with the average win probability of all non-adaptive tanks. The population of win probabilities per non-adapting tank contains the best performing and the worst performing tanks. Each non-adapting tank competes in multiple matches within a larger tournament. The research team partitions these into categories based on whether the non-adapting tank wins a majority of its matches.

This study demonstrates H2 by executing another tournament of 100 matches against all non-adaptive systems. This tournament uses the two-branch topology with the behavior tree instead of the one-branch topology. The study compares the win probability of the two-branch topology against the win probability of the one-branch agent.

The comparison of probabilities uses an approximation to a binomial distribution function [28]. The study does not reject H2 if the mean of the two-branch method lies within 95 percent probability as defined by a two sample T-Test.

## 5 Results

An initial set of 840 tournaments featuring each non-adapting tank battling each of the other non-adapting tanks was conducted. The term *group* defines 100 matches between an adaptive tank and a non-adaptive tank. This required 705,600 groups of tank battles, or 70,560,000 individual matches. The experiment required a cluster of 21 Ruby on Rails servers to generate results within a 5 month time span. This initial data also yields the average win probabilities for the population of all non-adapting tanks, and the average win probabilities for the population of all non-adapting tanks that win within a group of matches (also referred to as “best performing”).

Table 2 shows the average in probabilities for these two categories of win probabilities. It indicates a win rate of 50 percent since the experiment consists of every tank type battling against every other tank type. That win rate isn't efficacious for verifying H1 since it contains the poorest performing tanks as well as the best performing tanks. When only the best performing tanks are considered, it becomes a more meaningful metric.

Table 3 shows the win probability for each type of adaptive tank. There are 12 different types of adaptive tanks. Each one of these is defined by two adaptation times, and two error rates. The adaptation time indicates a point in time, measured in time steps, that the adaptation event will occur. The error rate represents the probability that an adaptive system will not identify its opponent. If an adaptive system does not identify its opponent then it chooses a random identity and adapts to compete against that. Previous research has shown that two-stage adaptation achieves better performance levels than a single adaptation stage [1], within an RRobots simulation context. Table 3 also shows the performance difference between the single and double branch architectures.

The results of the self-healing study differ from previous results documented in [1]. The self-healing experiments showed that the overall performance of the system was largely unaffected by the time that the second adaptation decision was made. The adaptive system changes its behavior the first time using a classifier that has a higher error rate than the second behavior selection. This shows an advantage to using the fastest classifier initially, even when slower classifier has a lower error rate.

The first-adaptation data in Table 3 is represented in Clip 4. This shows a six points in time, measured by time-steps, which represent when the initial adaptation occurred. The larger point represents a point with a larger standard deviation than the others. The standard deviation is calculated using the win percentages that have been rounded off. This means that the point has a slightly different win probability based on the final adaptation time. All of the standard deviations were quite small, so the size of the data circle is a qualitative representation of that value. A dashed trend line shows how system performance is decreasing based on the increasing time of initial adaptation.

The two-branch adaptive strategy uses the results of the non-adapting tank tournaments to create a statistical basis for adaptation. Table 4 shows the probability that a non-adapting tank will win against another non-adapting tank based solely on the self-healing strategy used. Table 4 ignores the effects of firing strategies, movement strategies, aiming strategies, and firepower levels. Previous work on adaptive systems has generated a data set containing only these data elements without the self-healing strategies.

When Bayes Rule is applied, Table 4 transforms into a behavior classifier table. The research effort adds a new type of adaptive tank to the experiment using this data. The new adaptive tank contains two decision branches. The first branch uses legacy data to adapt based on non-healing strategies. The second branch uses the data contained in Table 5 as a classifier, selecting an appropriate attacker strategy based on the assumed strategy of an opponent.

The new adapting tank makes an assumption about the identity of its opponent. The error rate of the experiment governs whether this assumption is correct. If the assumption is not correct then the new adapting tank picks a random healing strategy as its assumption. When adaptation occurs the system uses the self-healing strategy with the highest probability of success. Asterisks denote these values in Table 5.

## 6 Discussion

The Hypothesis H1 stated that the performance of the self-adaptive system would exceed the average performance of the non-adaptive system. This study defines performance as the ability for one type of agent to win against another in series of simulated combats, also called trials. Table 2 shows that the average performance of a non-adapting tank is 50 percent. The adaptive tank chooses its behaviors from a collection of behaviors used by the different non-adapting tanks. The adaptive tank uses two different parameters to govern its behavior, an adaption time and a probability to identify its opponent. Table 3 contains the results of the adaptive battles. The adaptive systems won at a rate of 80 percent, which outperformed the non-adaptive win rate. This does not reject H1 so the study accepts H1 as demonstrated.

Some of the non-adapting systems perform poorly overall. The study defines a poorly performing system as one lost more than 50 percent of their matches. When those poorly-performing systems are removed, the win average became 84 percent with a standard deviation of 15.8 percent. The average win probability of the adapting agent lies within a standard deviation of 33 of the best performing non-adapting systems. The adaptive system uses its behavior selection to achieve a performance level closely equivalent to the best performing non-adaptive systems. Inspecting Table 3 shows that the adaptive systems outperform the best non-adapting systems if they are able to execute an adaption prior to time of 1000. This is consistent with experiments performed in previous studies.

Hypothesis H2 stated that the branch topology of the tree of adaptation decisions will not affect the outcome of these matches. This study defines the branch topology in terms of how the decision behaviors are organized in the selection tree. The adaptive system changes at predetermined times by first identifying its opponent, then selecting a behavior from among a set of candidates. The study considered two behavior selection topologies.

The first topology consists of a single decision branch that selected a single behavior from a pool that contained every behavior found in the non-adaptive systems (found in Table 1). This contained 840 distinct choices.

The second topology consisted of two decision branches. The first branch selects a single behavior from a pool of all non-healing behaviors. This contained 210 behaviors. The second branch contained 4 behaviors based on the different self-healing strategies (found in Table 1). The adaptive system used both of these branches to select two candidate behaviors. It then merges these behavior selections into a single behavior set and used this newly merged behavior as its choice. The two-branch topology reduces the amount of system memory requirements to 25.47 percent of the memory used by the one-branch topology. Additional memory offers a system the capability to add additional behaviors to the selection process and thus improve performance, given a static hardware platform.

A quick analysis of branching strategy memory usage was performed using simplifying assumptions. This analysis comparing the distributions of these results is used to decide whether H2 is not rejected. The analysis assumes a record based memory structure where the size of the record is based on the size of the record fields using C++ language structures. These structures contain 32 bit integers that represent a strategy selection, and 64 bit pointer references that contain memory locations. These records reference the Firing, Damage, Movement, Aiming, and Healing strategies. The single branch architecture consumes 30 kB of memory. The two-branch method uses 7 kB of memory. If the approach were expanded to 5 branches, this would require only 4 kB of memory. The amount of memory available loosely correlates to the number of behaviors and decisions that an adaptive can perform. The study evaluates win probabilities based on trials between adaptive and non-adaptive systems. Table 3 contains the win probabilities for both topologies across each experiment. The study tests if the win rates from the one-branch topology and two-branch topology systems belong to the same distribution. This test uses Microsoft Excel to generate this metric using a paired sample T-Test. If the paired sample T Test value is less than 0.95 then the study rejects H2. The data generated a T-Test probability of 0.9852. The study does not reject H2.

This paper described building an adaptive self-healing system that exists within a resource-constrained environment. The system uses two different types of systems to identify appropriate behavior choices. The study further investigated the tradeoffs between different configurations of the behavior selection system. It implemented an adaptive self-healing system as a simulated tank that battles non-adapting tanks. The contest represents a generic adaptive systems problem where one system seeks to adapt in order to counter or overcome another system. This type of scenario features actions that are constrained by resources or limited system states within the shared environment. This study represented such restrictions in the form of battlefield position and boundaries along with the control of healing resources. This is a benefit to system engineers allowing them to design systems with many more behavior options within available memory constraints, or to scale their designs to smaller platforms.

The results of this study indicated that a system built using the tree-based NASP pattern outperformed the non-adaptive systems. These adaptive systems demonstrated sensitivity to the timing of adaptation. If a system adapted too late, then its adaptation did not greatly affect the outcome a tournament. When the adaptation occurred earlier in the tournament, the adaptive system performed equivalently or better than the best performing non-adaptive systems. This indicates that the design pattern is a useful starting point when designing a new adaptive system with temporal constraints.

The study also investigated how a branching structure within the adaptive system affected performance. Two structures were compared against each other. A single branch structure selected behaviors from among 840 candidate behaviors. A two-branch method selected behaviors from a set if 210 candidate behaviors. The performance of the two types of adaptive systems was essentially the same. This result indicates that additional behaviors and behavior selection mechanisms defined in the NASP do not adversely affect system performance. Instead, they improve the overall spatial efficiency of the adaptive system.

The NASP design pattern has demonstrated usefulness when creating adaptive systems based on Bayesian classifiers. The research team feels that this approach is sufficiently generic so that it will support other types of classification algorithms without changing the overall pattern. In the future, the team will investigate using Neural Networks [29] or Genetic Algorithms [30] within a NASP based decision tree.

## Declarations

### Author contribution statement

Brian Phillips: Conceived and designed the experiments; Performed the experiments; Analyzed and interpreted the data; Contributed reagents, materials, analysis tools or data; Wrote the paper.

Mark Blackburn: Conceived and designed the experiments; Wrote the paper.

### Competing interest statement

The authors declare no conflict of interest.

### Funding statement

The authors received no funding from an external source.

### Additional information

Data associated with this study has been deposited at https://bitbucket.org/blackburnphd/railsrobots/.

## Figures and Tables

**Clip 1:**
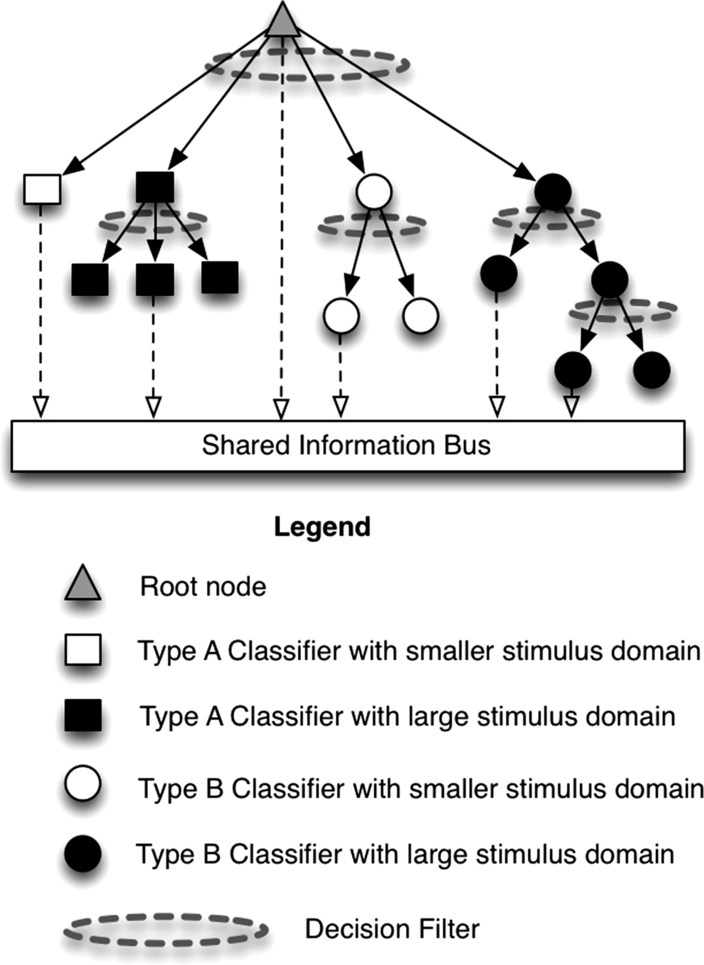
NASP component architecture.

**Clip 2:**
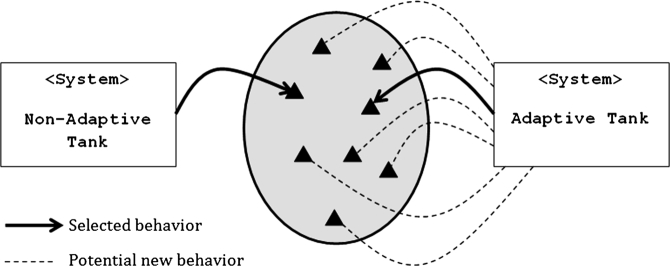
Behavior choice.

**Clip 3:**
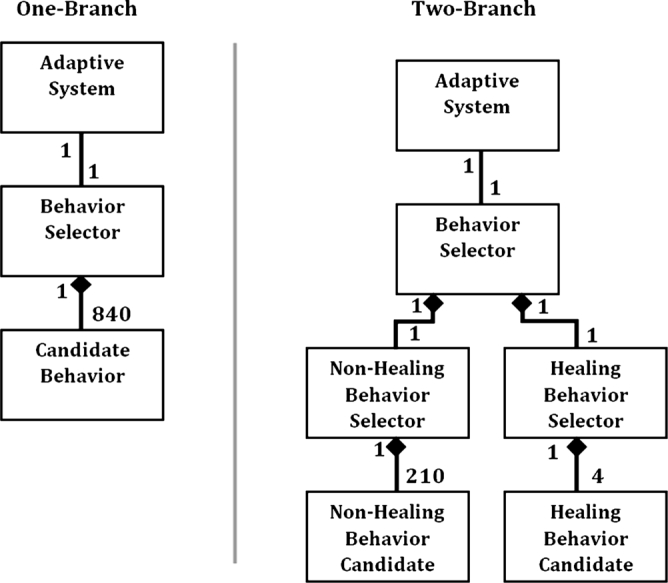
Comparing two decision topologies.

**Clip 4:**
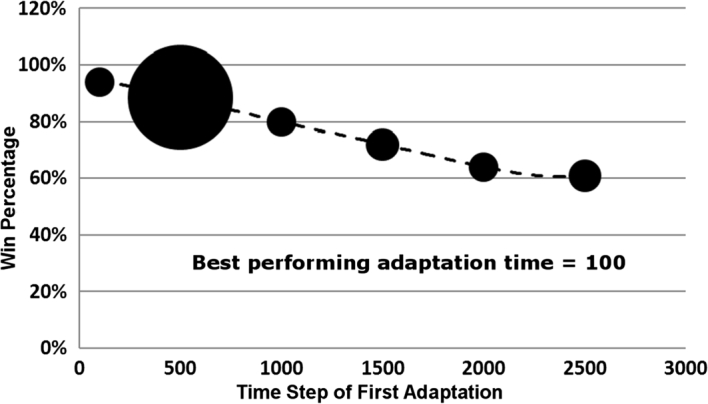
First adaptation time governs system performance for self-healing case.

**Table 1:** Simulation configuration.

Parameter	Value
Firing strategy	0 – Always shoot based on damage
	1 – Change damage level based on distance
	2 – Use a damage level of 1.0 when sensors have previously detected the opponent

Damage level	0.1, 0.6, 1.0, 1.1, 1.6, 2.1, 2.6 energy points

Movement strategy	0 – Move in a circle
	1 – Move in a circle, when hit, change circular pattern
	2 – Maintain a fixed location
	3 – Move along the boundaries of the battlefield
	4 – When an opponent is detected, move directly toward it

Aiming strategy	0 – Attempt to anticipate where moving opponent will be
	1 – Shoot across a wide angle with a rotating gun

Healing strategy	0 – Ignore Cargo and Helicopter, continue battle
	1 – Move to the Cargo when it arrives
	2 – Attack Cargo
	3 – Attack Helicopter

**Table 2:** Average non-adapting tank win probability.

Tank type	Win probability	Std deviation
All non-adapting tanks	50 percent	37.30
Best performing tanks	84 percent	15.8

**Table 3:** Adaptive system win probability.

Adaptation time		Win probability	
First	Second	One adaptation	Two adaptations
100	1000	94 percent	94 percent
100	2000	94 percent	94 percent
100	3000	94 percent	94 percent
500	1000	89 percent	89 percent
500	2000	89 percent	89 percent
500	3000	88 percent	89 percent
1000	2000	80 percent	80 percent
1000	3000	80 percent	80 percent
1500	2000	72 percent	72 percent
1500	3000	72 percent	72 percent
2000	3000	64 percent	66 percent
2500	3000	61 percent	60 percent

**Table 4:** Win probability based on heal strategy.

Tank-1 strategy	Tank-2 strategy			
0 – Ignore	1 – Pick up Cargo	2 – Attack Cargo	3 – Attack Helicopter
0 – Ignore	50 percent	39 percent	54 percent	64 percent
1 – Pick up Cargo	61 percent	49 percent	60 percent	66 percent
2 – Attack Cargo	45 percent	40 percent	49 percent	62 percent
3 – Attack Helicopter	36 percent	34 percent	37 percent	51 percent

**Table 5:** Bayesian behavior classifier.

Tank-1 strategy	Tank-2 strategy			
0 – Ignore	1 – Pick up Cargo	2 – Attack Cargo	3 – Attack Helicopter
0 – Ignore	26 percent	2 percent	28 percent	*27 percent
1 – Pick up Cargo	*34 percent	*31 percent	*30 percent	25 percent
2 – Attack Cargo	22 percent	25 percent	25 percent	26 percent
3 – Attack Helicopter	18 percent	22 percent	17 percent	22 percent

## References

[1] Phillips B.J., Blackburn M. (2014). Experimental trials based on a neocortex-based adaptive system pattern. Proc. Comput. Sci..

[2] Salehie M., Tahvildari L. (2009). Self-adaptive software: landscape and research challenges. ACM Trans. Auton. Adapt. Syst. (TAAS).

[3] Cheng B.H.C., Lemos R., Giese H., Inverardi P., Magee J., Andersson J., Becker B., Bencomo N., Brun Y., Cukic B., Marzo Serugendo G., Dustdar S., Finkelstein A., Gacek C., Geihs K., Grassi V., Karsai G., Kienle H.M., Kramer J., Litoiu M., Malek S., Mirandola R., Müller H.A., Park S., Shaw M., Tichy M., Tivoli M., Weyns D., Whittle J., Hutchison D., Kanade T., Kittler J., Kleinberg J.M., Mattern F., Mitchell J.C., Naor M., Nierstrasz O., Pandu Rangan C., Steffen B., Sudan M., Terzopoulos D., Tygar D., Vardi M.Y., Weikum G., Cheng B.H.C., Lemos R., Giese H., Inverardi P., Magee J. (2009). Software engineering for self-adaptive systems: a research roadmap. Software Engineering for Self-adaptive Systems.

[4] IBM (2005). An architectural blueprint for autonomic computing. http://www-03.ibm.com/autonomic/pdfs/ACBlueprintWhitPaperV7.pdf.

[5] Dabrowski C., Mills K. (2002). Understanding self-healing in service-discovery systems.

[6] Sheng S., Li K., Chan W., Xiangjun Z., Xianzhong D. (2006). Agent-based self-healing protection system. IEEE Trans. Power Deliv..

[7] Shapiro M.W. (2004). Self-healing in modern operating systems. Queue.

[8] Garlan D., Schmerl B. (2002). Model-based adaptation for self-healing systems.

[9] Havelund K. (2011). Implementing runtime monitors. TORRENTS 2011, 2nd TORRENTS Workshop.

[10] Minsky N. (2003). On conditions for self-healing in distributed software systems. IEEE Comput. Soc..

[11] George S., Evans D., Davidson L. (2002). A biologically inspired programming model for self-healing systems.

[12] Greensmith J., Whitbrook A., Aickelin U., Gendreau M., Potvin J.-Y. (2010). Artificial immune systems. Handbook of Metaheuristics.

[13] Siljee J., Bosloper I., Nijhuis J., Hammer D., Hutchison D., Kanade T., Kittler J., Kleinberg J.M., Mattern F., Mitchell J.C., Naor M., Nierstrasz O., Pandu Rangan C., Steffen B., Sudan M., Terzopoulos D., Tygar D., Vardi M.Y., Weikum G., Benatallah B., Casati F., Traverso P. (2005). DySOA: making service systems self-adaptive. Service-oriented Computing – ICSOC 2005.

[14] United States Department of the Air Force (2014). Capabilities for cyber resiliency. https://www.fbo.gov/index?s=opportunity&mode=form&id=d2a95b03a8621c1be03128e02f10d66a&tab=core&_cview=0.

[15] Judy J.W. (2012). Neural interfaces for upper-limb prosthesis control: opportunities to improve long-term reliability. IEEE Pulse.

[16] Saha G.K. (2007). Software – implemented self-healing system. CLEI Electron. J..

[17] Sokolsky O., Rosu G. (2012). Introduction to the special issue on runtime verification. Form. Methods Syst. Des..

[18] Aickelin U., Dasgupta D., Gu F., Burke E.K., Kendall G. (2014). Artificial immune systems. Search Methodologies.

[19] Ghosh D., Sharman R., Raghav Rao H., Upadhyaya S. (2007). Self-healing systems — survey and synthesis. Decis. Support Syst..

[20] Gamma E. (1995). Design Patterns: Elements of Reusable Object-oriented Software.

[21] Phillips B., Blackburn M. (2014). Towards a design pattern for adaptive systems inspired by the physical architecture of the neocortex. 4th International Engineering Systems Symposium.

[22] Salakhutdinov R., Tenenbaum J.B., Torralba A. (2013). Learning with hierarchical-deep models. IEEE Trans. Pattern Anal. Mach. Intell..

[23] Kahneman D. (2013). Thinking, Fast and Slow.

[24] Hubel D.H. (1988). Eye, Brain, and Vision.

[25] Lee T.S., Mumford D. (2003). Hierarchical Bayesian inference in the visual cortex. J. Opt. Soc. Am..

[26] Felleman D.J., Van Essen D.C. (1991). Distributed hierarchical processing in the primate cerebral cortex. Cereb. Cortex.

[27] Corkill D. (1991). Blackboard systems. AI Expert.

[28] Wallis S. (2013). Binomial confidence intervals and contingency tests: mathematical fundamentals and the evaluation of alternative methods. J. Quant. Linguist..

[29] Rosenblatt F. (1958). The perceptron: a probabilistic model for information storage and organization in the brain. Psychol. Rev..

[30] Holland J.H., Reitman J.S. (1977). Cognitive systems based on adaptive algorithms. ACM SIGART Bull..

